# Supplementary material for: Transcription factor network analysis identifies REST/NRSF as an intrinsic regulator of CNS regeneration in mice
Source: Nat Commun. 2022 Jul 29;13:4418. doi: 10.1038/s41467-022-31960-7 (PMC9338053; doi:10.1038/s41467-022-31960-7)
Supplement: Supplementary file 1 — Supplementary Information [file 41467_2022_31960_MOESM1_ESM.pdf]

Supplementary Fig. 1

a

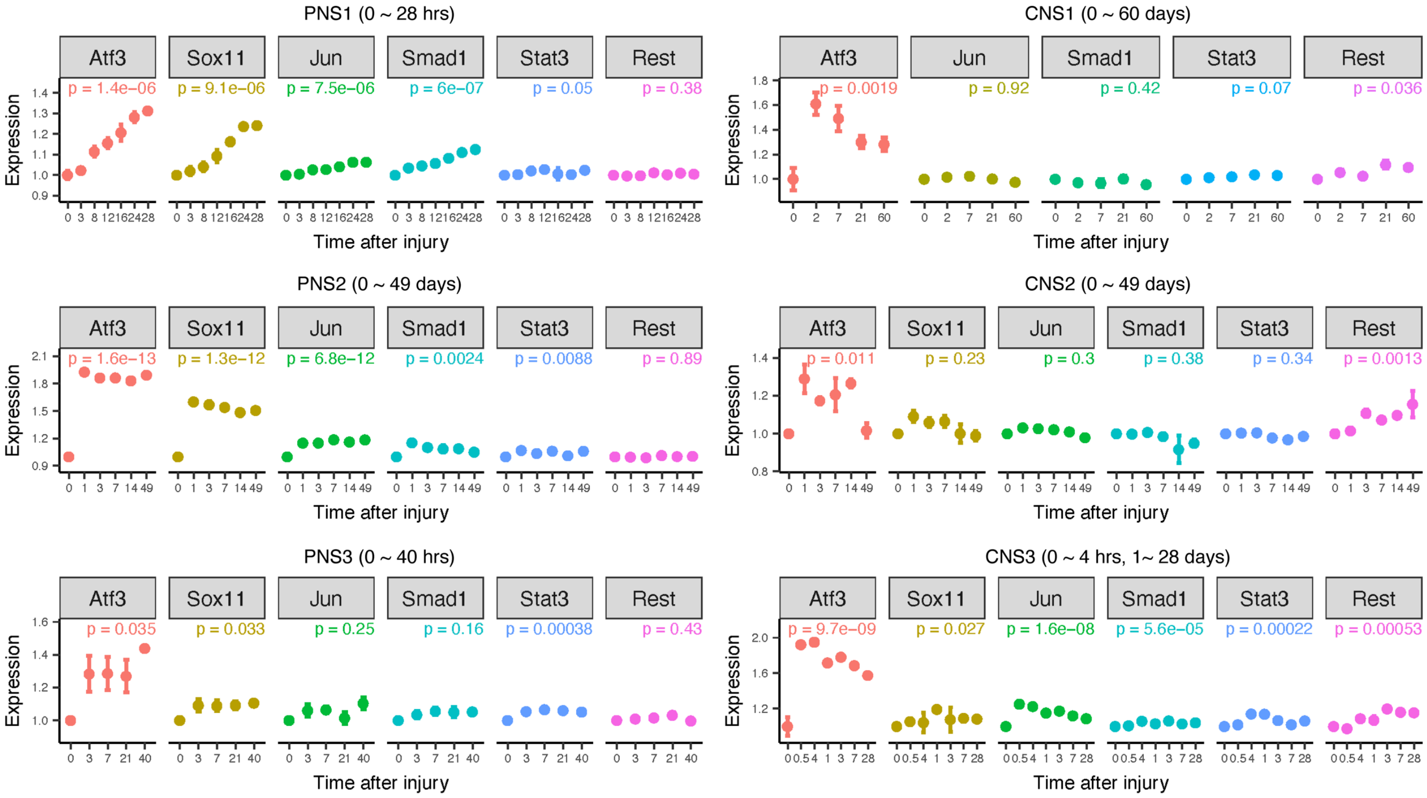

Supplementary Fig. 1. TF mRNA expression levels following PNS or CNS injury, related to Figure 2. (a) Relative expression levels of key regenerative TFs (*Atf3, Sox11, Jun, Smad1, Stat3, Rest*) across multiple PNS and CNS injury datasets listed in Figure 2B. Each dot represents the mean log fold changes normalized to control sham condition. N = 3 in each time point and error bars indicates SEM. Two-side p-values are from ANOVA comparing injured with sham control samples, adjusted with TukeyHSD.

Supplementary Fig. 2

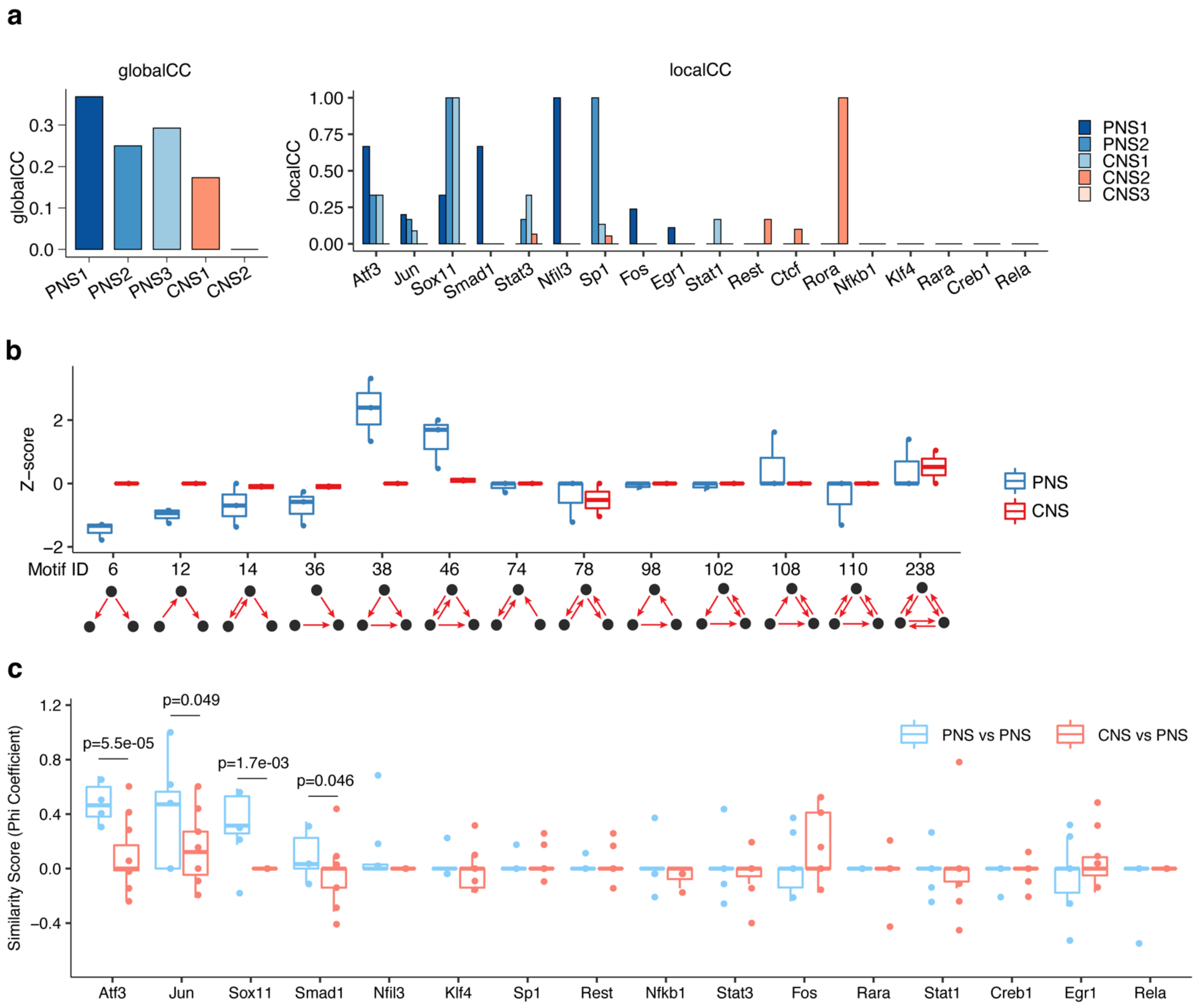

Supplementary Fig. 2. TF networks comparing PNS and CNS, related to Figure 2. (a) TF network statistics including global and local clustering coefficient. Global clustering coefficient (globalCC) was calculated for each network graph, as the number of closed triangles formed by 3 vertexes over the total number of triangles (open or closed) in the graph. A high global clustering coefficient indicates the overall graph connectivity. Local clustering efficient (localCC) is calculated for each TF vertex, as the number of links between the vertices within its neighborhood divided by the number of links that are possible between them. A high local clustering coefficient suggest a tightly connected local network. **(b)** Over- (Z-score > 0) or under-representation (Z-score < 0) of each 3-node motif in a PNS or CNS network. The motif ID is the decimal format of the network motif. For example, the feedforward-loop id is 38 (binary 011001000, with least significant bit on the left). **(c)** Similarity of ARACNe-identified TF networks across multiple PNS and CNS injury datasets listed in Figure 2B. Phi coefficient was used to calculate the correlation of each TF's networks between two datasets since a TF's connections with the other TFs are binary (1 means a connection exists between two TFs and 0 means no connection). Each dot represents the Phi coefficient of a TF network between two datasets. Blue dots indicate similarity of TF networks comparing PNS vs PNS, and red dots represent similarity comparing CNS vs PNS. Five PNS and three CNS datasets were used for comparison. The box ranges from the first quartile to the third quartile of the distribution, the line represents the median, and the whiskers represents the minimum (lower) or maximum (upper) values. Statistical differences were calculated by Welch two sample t-test on Fisher-transformed z values from Phi coefficient, as coefficient correlations are not normally distributed. Comparisons with no statistical difference ($p > 0.05$) were not labeled with its p-values.

Supplementary Fig. 3

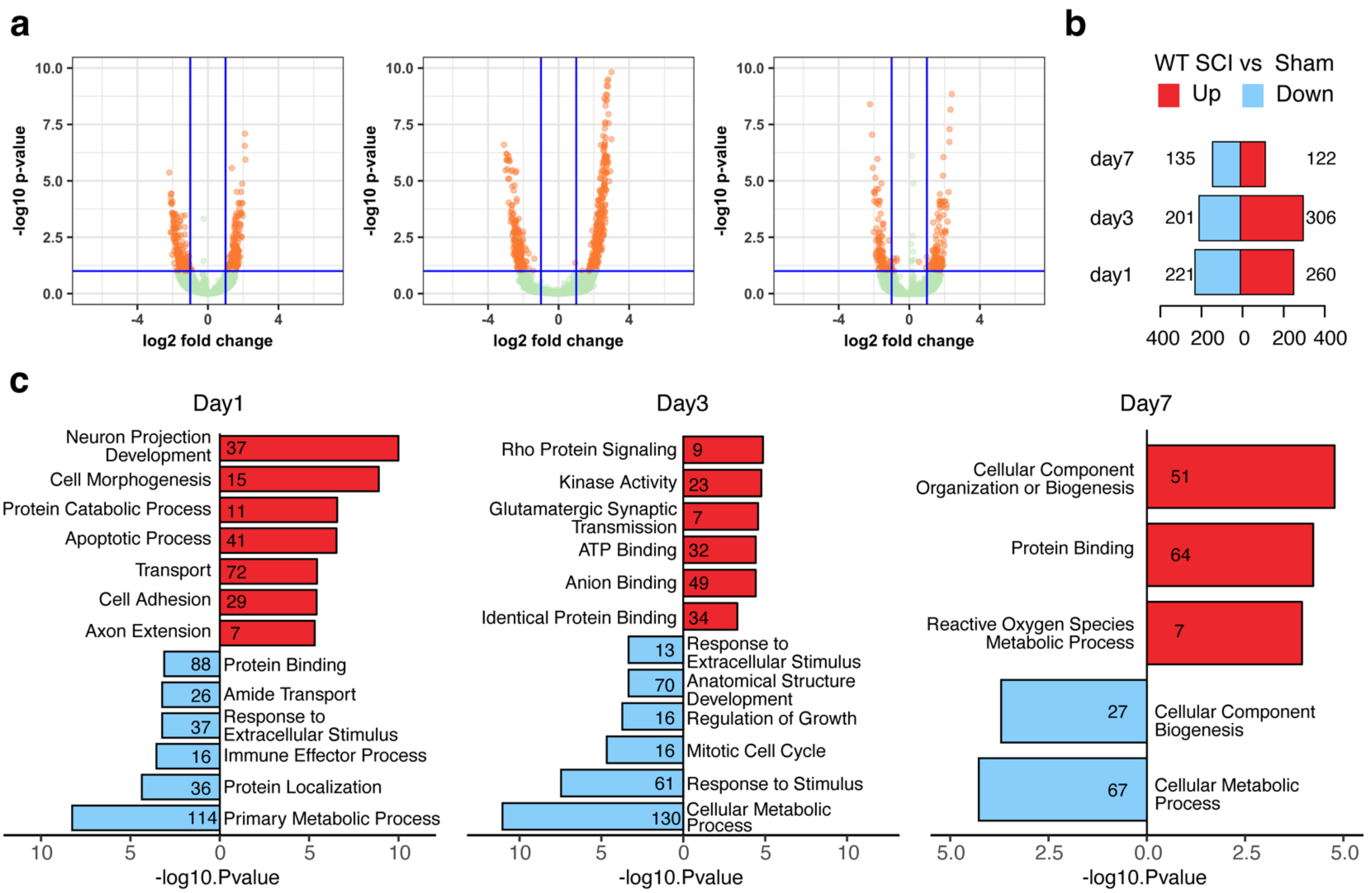

Supplementary Fig. 3. Differential gene expression analysis on RNA-seq of cortical neurons at 1, 3 and 7 days following SCI, related to Figure 3. (a) Volcano plots showing differentially expressed genes (DEGs) at FDR-corrected, two-side p value < 0.1 at 1, 3, 7 and days after SCI compared to sham-treated group. **(b)** Number of DEGs at each time point. Up-regulated: red; Down-regulated genes: blue; $|\log2\ FC| > 0.3$). **(c)** Top gene ontology (GO) terms associated with DEGs at indicated condition (FDR adjusted one-side enrichment p-value). A full list of DEGs are listed in Supplementary Data 1.

Supplementary Fig. 4

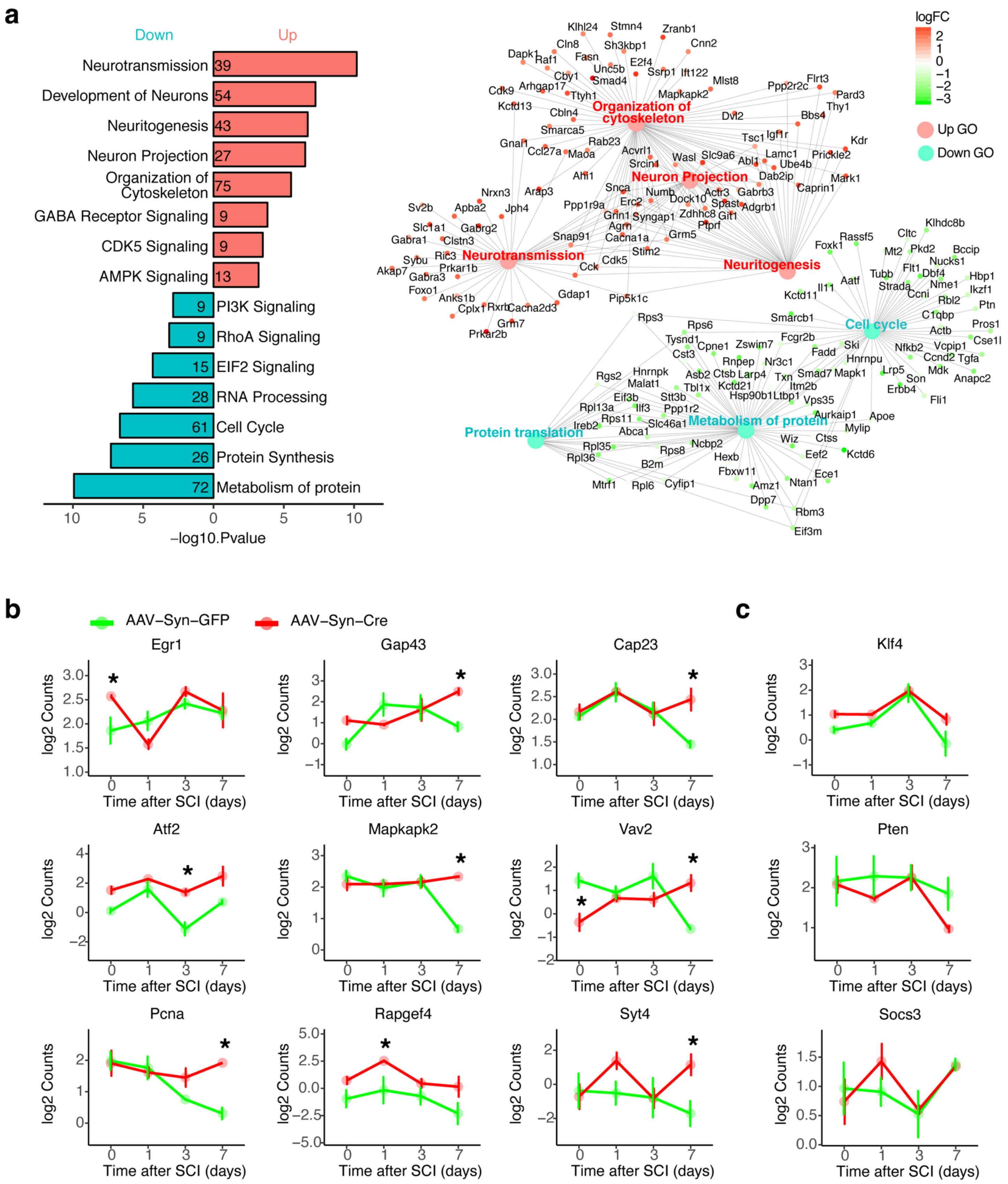

Supplementary Fig. 4. Differential gene expression analysis on RNA-seq of injured cortical neurons comparing wild-type and REST-depleted mice, related to Figure 3. (a) Top gene ontology (GO) terms associated with DEGs (FDR-corrected one-sdie p value < 0.1) comparing sensorimotor cortical neurons with or without REST depletion at day 7 following SCI. Bars represent FDR adjusted enrichment p-value in the negative log scale. Numbers in the bars indicate the number of DEGs overlapping existing in each GO term. The network plot shows DEGs enriched in each of the top up- or down-regulated GO terms. Colors indicates logFC of these genes comparing REST knock-out neurons to wild-type controls. **(b-c)** Expression levels of regeneration associated transcription factors (TFs) and genes comparing wild-type with REST knock-out cortical neurons recovered following SCI. Values are mean log2 Counts ± SEM from the RNA-seq data. n = 3 - 4 mice in each condition. Asterisks denotes FDR-corrected, two-tail P < 0.1 compared to AAV-Syn-GFP at each time point. Exact P values and n numbers are included in Supplementary Table 1.

Supplementary Fig. 5

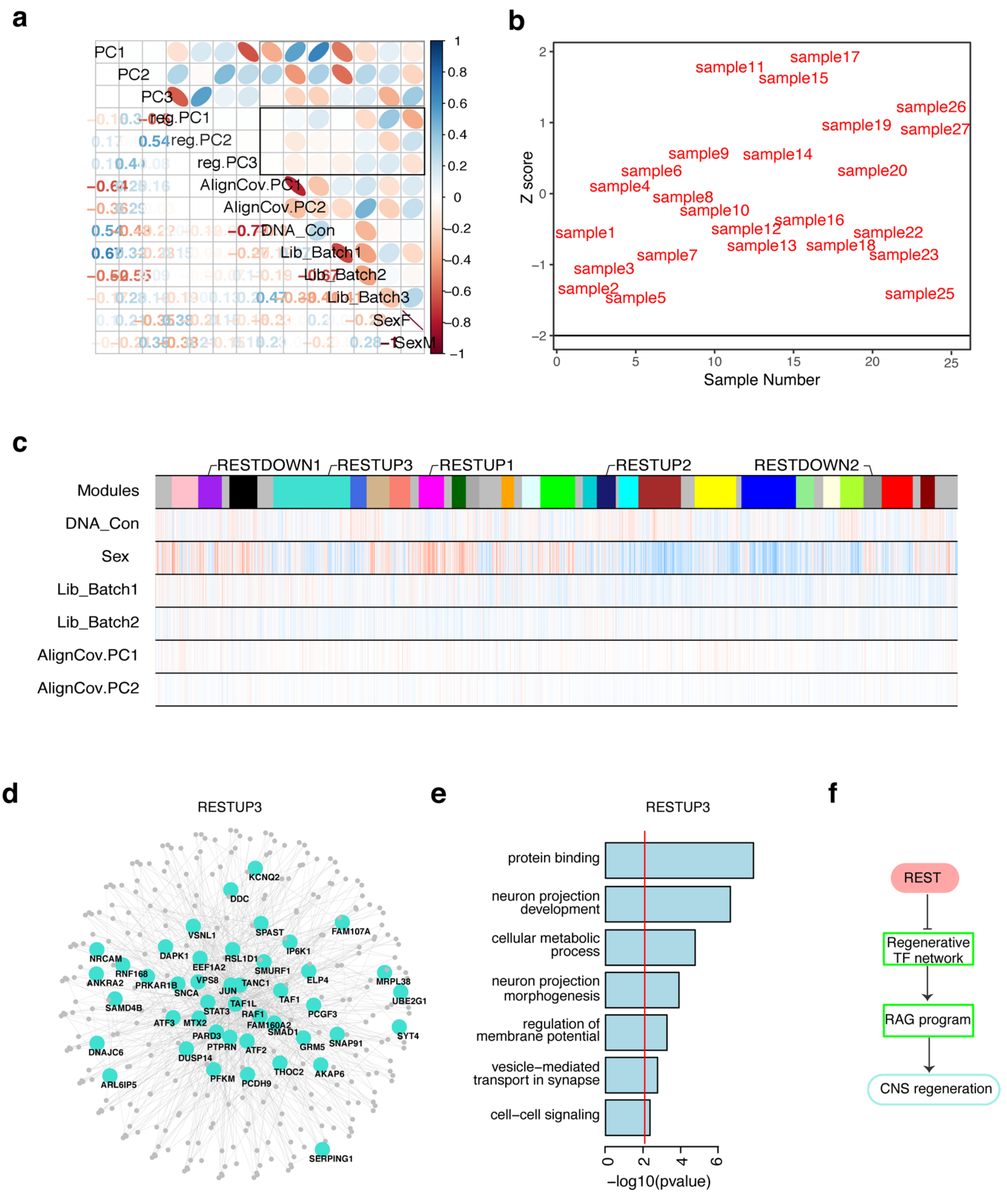

Supplementary Fig. 5. WGCNA analysis on RNA-seq of wild-type or REST knockout cortical motor neurons recovered following SCI, related to Figure 3. (a) Correlations between the first three principle components (PCs) of gene expression data with sex, batch, library concentration and sequencing bias, before or after (highlighted by the black square) linear regression of these covariates from the RNA-seq data. **(b)** Sample connectivity to determine outliers and samples with |Z-score| < 2 were removed. **(c)** WGCNA module correlations with covariates. MEs and gene membership in each module (kME) are in Supplementary Data 3. **(d)** PPI network of RESTUP3 module. The top 70 hub genes which represent the most central genes in the RESTUP3 module were labeled in the network plot. **(e)** GO terms associated with RESTUP3 module. Bars represent -log10(FDR-corrected one-side p-values). **(f)** A hypothetical model of how REST acts on CNS axon regeneration.

Supplementary Fig. 6

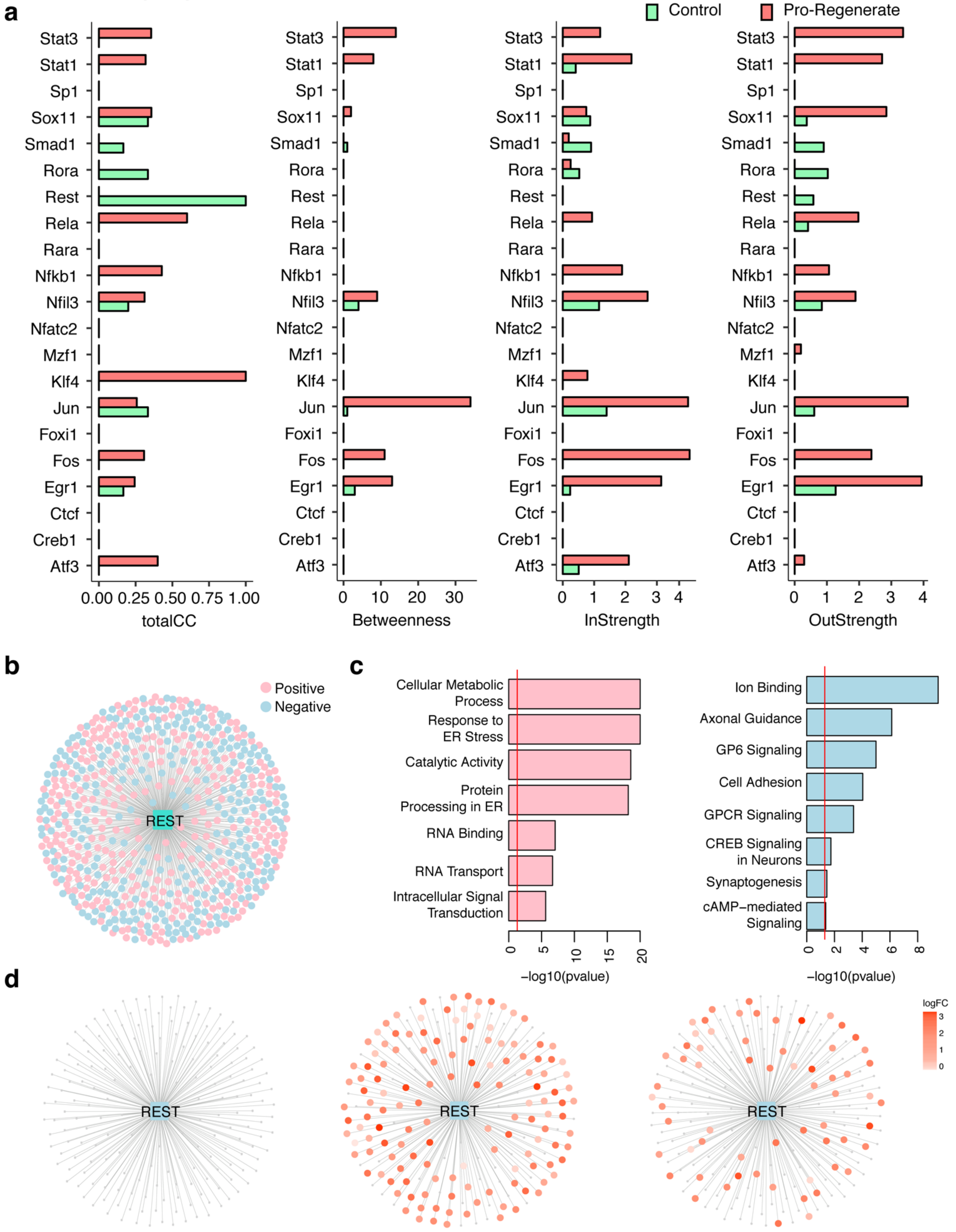

Supplementary Fig. 6. TF networks comparing injured RGCs with pro-regenerative treatments or non-regenerative control, related to Figure 4. TF networks were constructed from RNA-seq of RGCs sorted at 1, 3, 5 days after optic nerve crush alone (non-regenerating control) or with pro-regenerative treatment (AAV2-shPten.mCherry/Oncomodulin/CPT-cAMP; pro-regenerative), using the unbiased, step-wise pipeline described in Figure 2A. **(a)** Parameters indicating connectivity of each TF in the networks of pro-regenerative RGCs and non-regenerating control RGCs. Local clustering coefficient (totalCC): the clustering coefficient of a vertex which is calculated as the number of links between the vertices within its neighborhood divided by the number of links that are possible between them. A high local clustering coefficient suggest a tightly connected local network. Betweenness: the fraction of the shortest paths between all pairs of vertices that pass through one vertex. InStrength or OutStrength: the number of links connected to one vertex. In directed and weighted networks, the number of arcs that end at the node is defined as "InStrenght", and the number of arcs that start from the node is defined as "OutStrength". **(b)** Genes that are positively regulated (activated, pink-colored nodes) or negatively regulated (repressed, blue-colored nodes) by REST defined by ARACNe from RNA-seq of RGCs sorted at 1, 3, 5 days after optic nerve crush alone or with pro-regenerative treatment. FDR adjusted p value < 0.05, permutations = 100, bootstrap consensus = 95% were used to identify REST-regulated genes by ARACNe. **(c)** Top GO terms of REST-activated genes and REST-repressed genes. Bars represent -log10(FDR-corrected one-side p-values). **(d)** Overlap between REST-repressed genes and up-regulated genes by pro-regenerative treatment in RGCs at day 3 and day 5 following optic nerve crush. Colors represent logFC of significantly up-regulated genes by pro-regenerative treatments determined by FDR < 0.1. A full list of REST-repressed genes is shown in Supplementary Data 4.

Supplementary Fig. 7

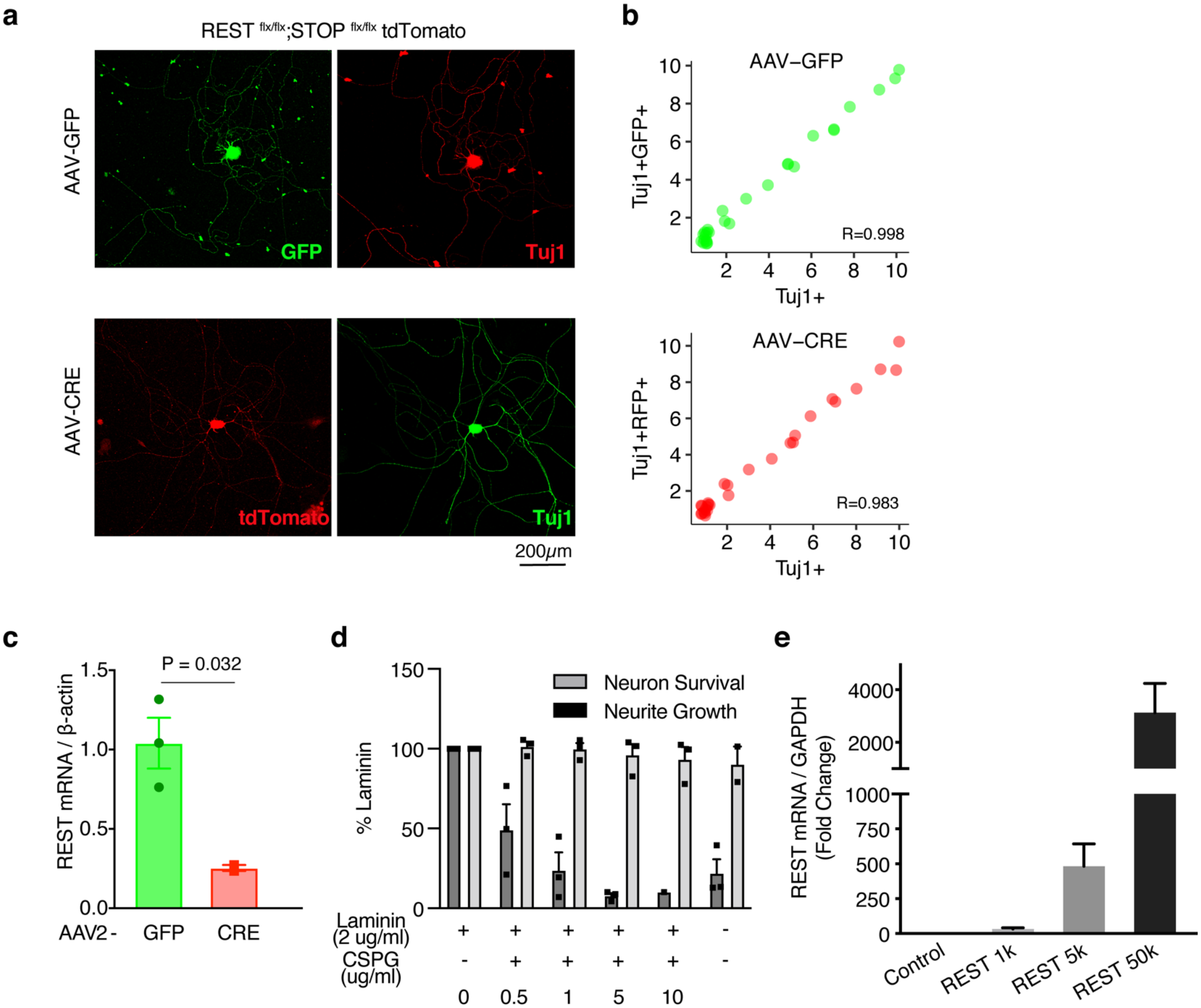

Supplementary Fig. 7. Validation of REST overexpression or knockdown in vitro, related to Figure 6. REST $^{flx/flx}$;tdTomato DRG cells were transduced with AAV-GFP or AAV-CRE for 7 days. **(a)** Confocal images of GFP or tdTomato co-stained with the neuronal marker Tuj1 to confirm efficient AAV transduction into DRG neurons as well as Cre-induced tdTomato expression, an indication of REST deletion. **(b)** A Pearson correlation between the number of Tuj-expressing neurons (Tuj1+) and the ones also expressing GFP (Tuj1+GFP+; AAV-GFP transduced neurons) or tdTomato (Tuj1+RFP+; AAV-CRE transduced neurons). Each dot represents an individual image quantified. A high correlation suggests efficient transduction of AAVs into the DRG neurons. **(c)** qRT-PCR of REST mRNA levels to confirm REST knockdown in DRG cultures. **(d)** Neurite outgrowth and neuronal survival at indicated doses of CSPG to determine an optimal dose of CSPG used in Figure 6A. **(e)** qRT-PCR of REST mRNA levels in DRG neurons to confirm REST overexpression by transducing the lentiviral constructs containing REST. Humanized luciferase protein (Lv135-hLuc) was used as a control. **(c-e)** Bars represents means±SEM; N = 3 replicate wells in each condition examined over 3 independent experiments. Statistical significance was assessed by two-tailed t-test.

Supplementary Fig. 8

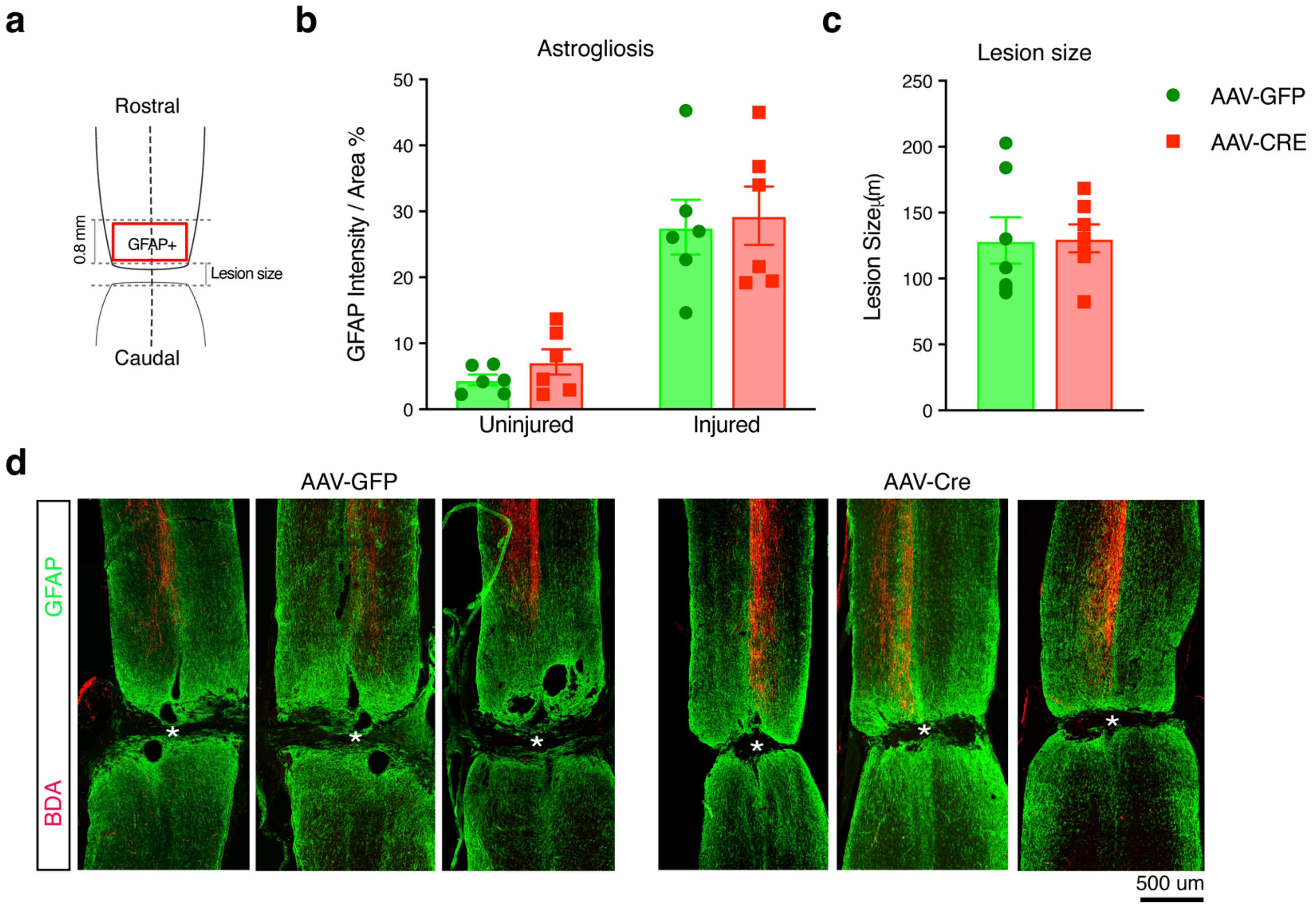

Supplementary Fig. 8. Quantitation of spinal cord injuries, related to Figure 7. (a) Schematic diagram showing regions for quantifying astrocyte activity and lesion size. **(b)** Quantitation of GFAP signals at squares (0.8 mm in width) drawn in the white matter of spinal cord sections close to the lesion center. **(c)** Lesion size quantified as the width of squares drawn covering all possible lesioned regions on spinal cord sections. **(b-c)** Bars represent mean ± SEM; N = 6 mice in each condition; Statistical significance was assessed by Student's t-test compared to AAV-GFP. **(d)** Randomly-chosen confocal images of BDA-labeled CST axons of lesioned spinal cord also stained for astrocytes.

Supplementary Fig. 9

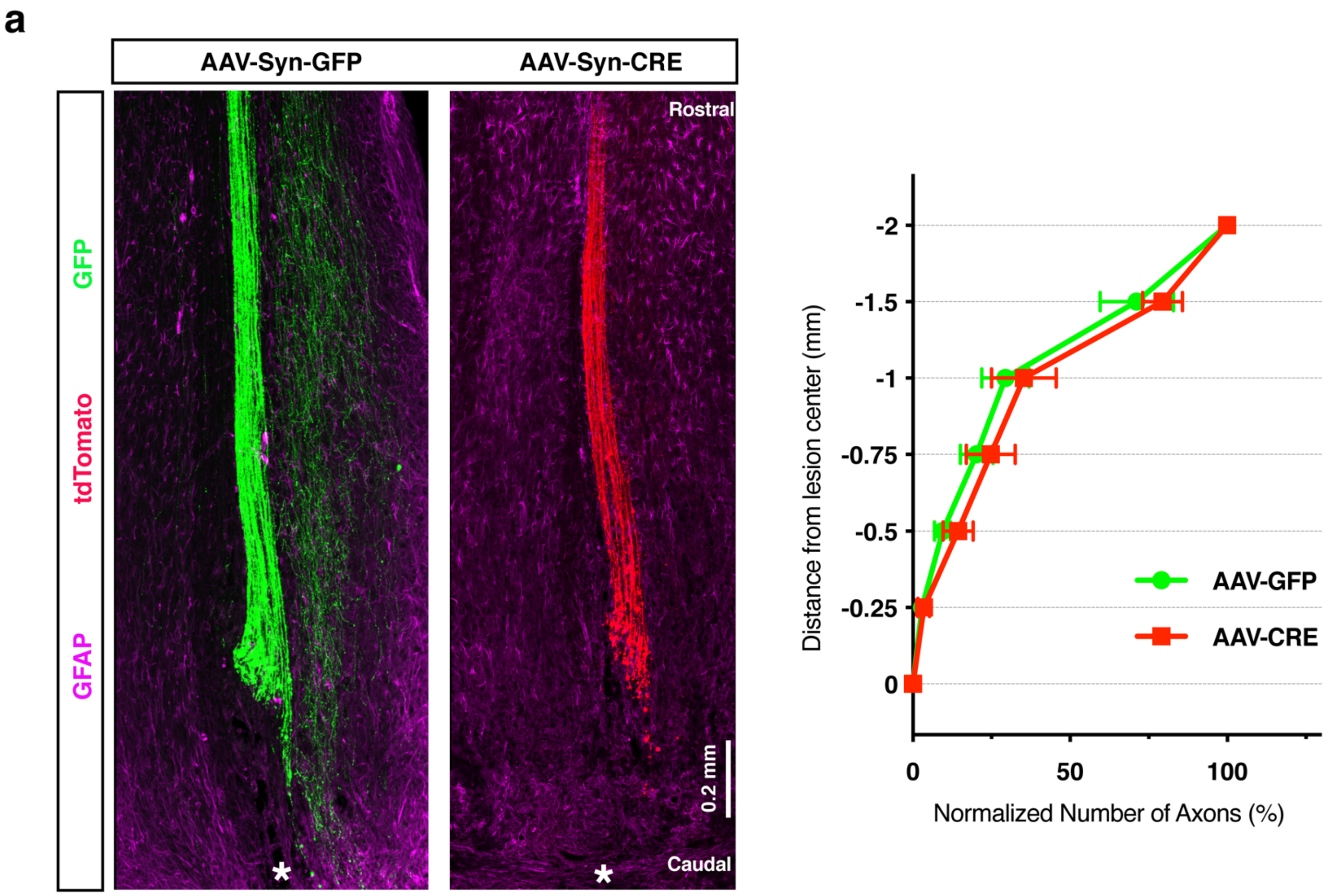

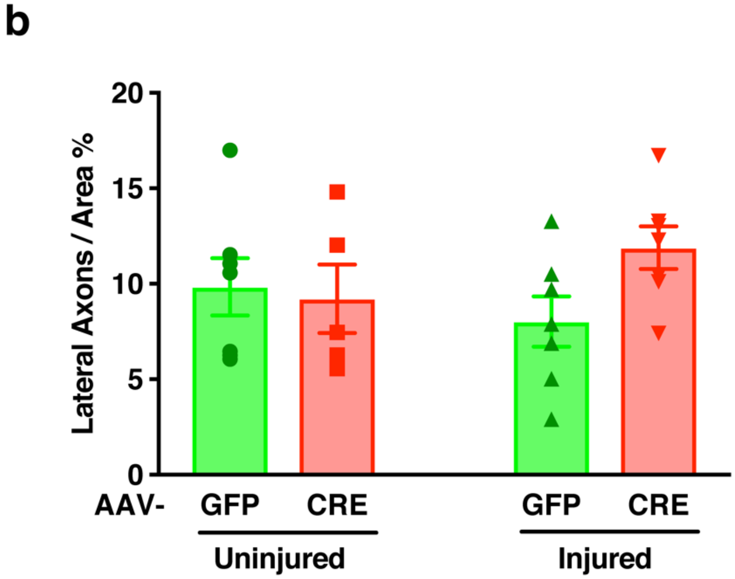

Supplementary Fig. 9. Corticospinal (CST) axon staining 3 days after spinal cord injury or in non-injured conditions, related to Figure 7. (a) Confocal images and quantitation of GFP or tdTomato labeled axons in horizontal sections of lesioned spinal cord also stained for astrocytes (glial fibrillary acidic protein (GFAP)). REST $^{flx/flx}$;tdTomato mice were injected into the sensorimotor cortex with AAV-Syn-GFP (wild-type) and AAV-Syn-CRE (REST cKO) for 4 weeks followed by a full crush at thoracic spinal cord level 10 (T10). Spinal cord was recovered 3 days post-injury. Lesion center was marked with *. To quantify axon numbers at indicated distances from lesion center, N=4 mice for each group was used. Bars represent means±SEM; no statistical difference was found between AAV-Syn-GFP with AAV-Syn-Cre using two-way ANOVA followed by Sidak. **(b)** Quantitation of total number of BDA-labeled axons in uninjured, or injured REST $^{flx/flx}$ mice receiving AAV-GFP or AAV-CRE. The paradigm for AAV transduction, spinal cord injury and BDA labeling was the same as described in Figure 7, except that SCI was not performed on uninjured mice. Total number of BDA-labeled axons in injured mice was counted 3 mm rostral to the lesion center. Bars represent means±SEM; N = 6 mice in each condition; No statistical difference was found by one-way ANOVA with Sidak post-hoc test compared to uninjured AAV-GFP.

Supplementary Fig. 10

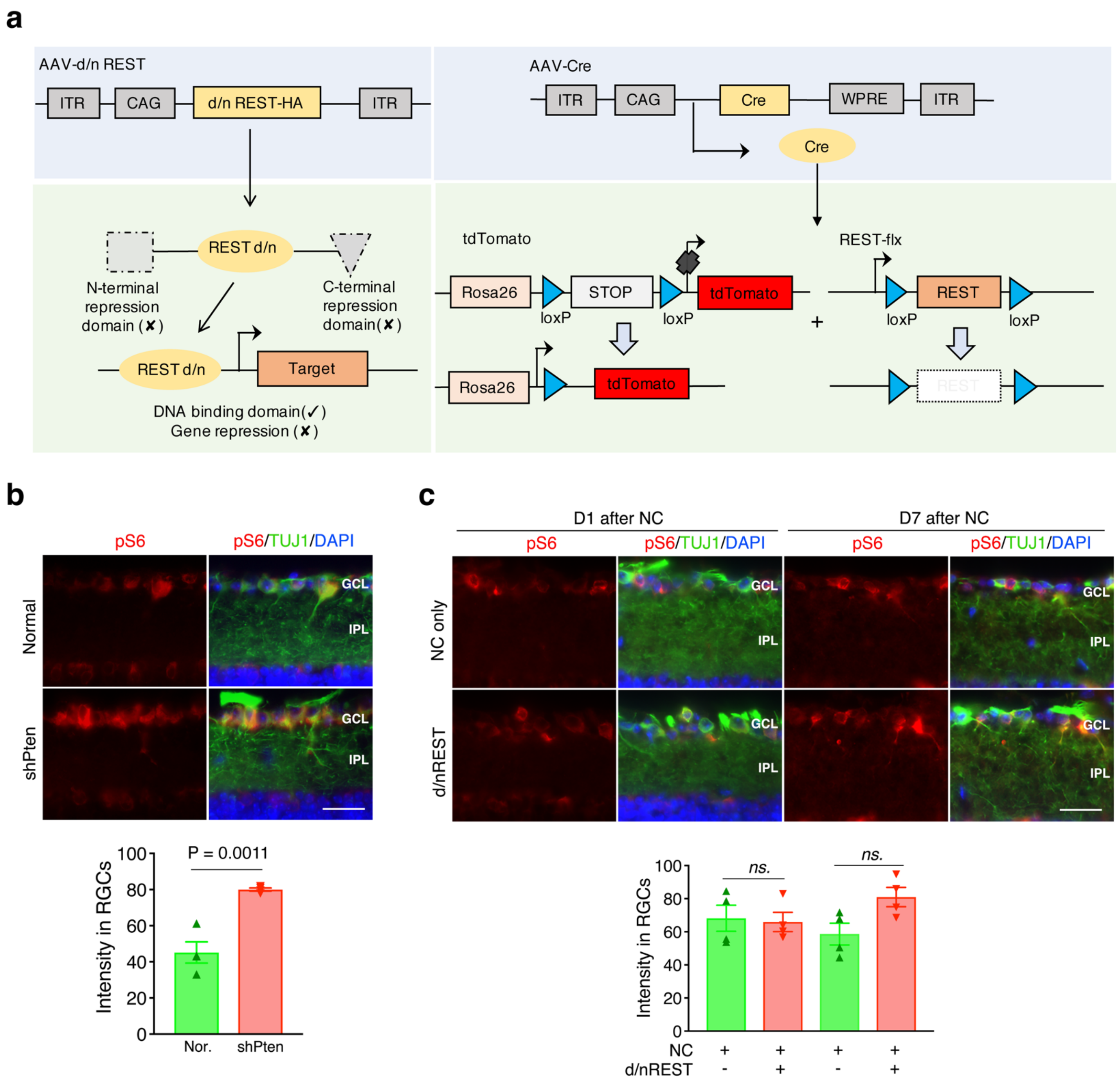

Supplementary Fig. 10. Conditional depletion or inhibition of REST in the model of optic nerve injury, related to Figure 8. (a) Schematic displaying strategies to induce REST inhibition or REST depletion in retina cells. **(b-c**) Inactivation of PTEN or REST initiates different downstream signaling pathways. Changes of phospho-S6 immunostaining (pS6, red) in RGCs (visualized by immunostaining for b**III** tubulin, green) after AAV2- mediated knock-down of PTEN (shPten) after optic nerve crush (NC), comparing with normal retina (Nor.) **(b)** or expression of dominant-negative REST (d/nREST) **(c)**. Whereas knockdown of PTEN elevated levels of p-S6 **(b)**, REST deletion did not (**c)**: expression shown at 1 and 7 days after nerve crush). **(B)** n= 3 mice in each condition, tested with two-tailed *t*-test. **(c)** n = 4 mice in each condition, tested with one-way ANOVA followed by Sidak test. Scale bars: 45 µm. Bars represent Mean ± SEM.

Supplementary Fig. 11

a

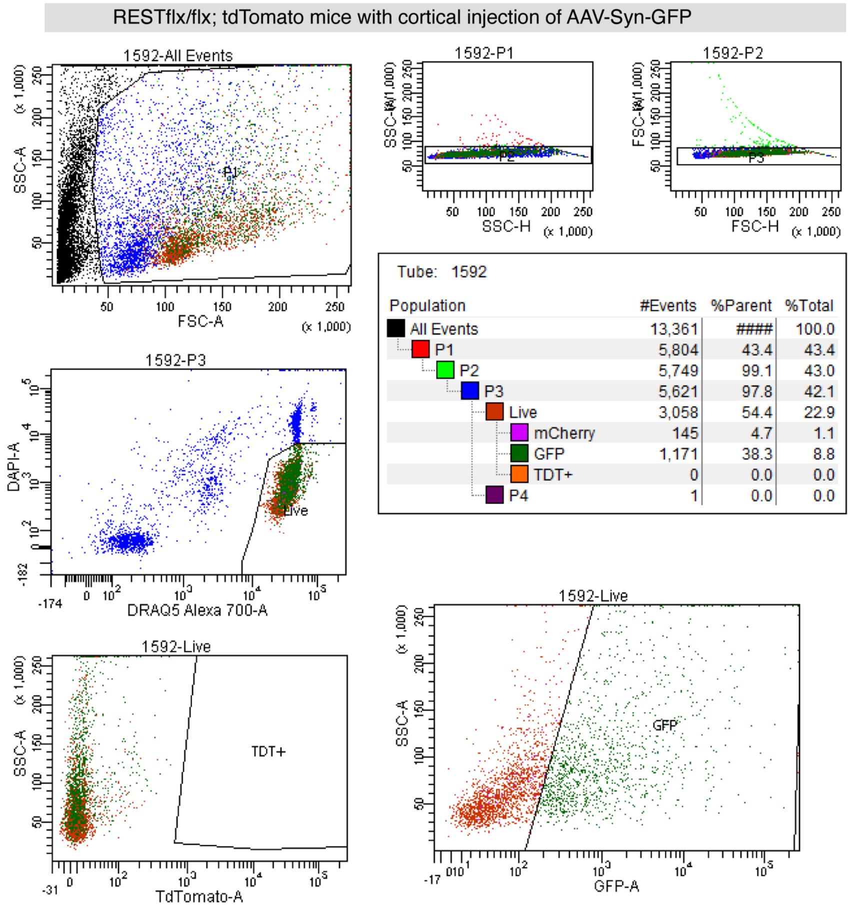

Tube: 1592

Population	#Events	%Parent	%Total
All Events	13,361	####	100.0
P1	5,804	43.4	43.4
P2	5,749	99.1	43.0
P3	5,621	97.8	42.1
Live	3,058	54.4	22.9
mCherry	145	4.7	1.1
GFP	1,171	38.3	8.8
TDT+	0	0.0	0.0
P4	1	0.0	0.0

b

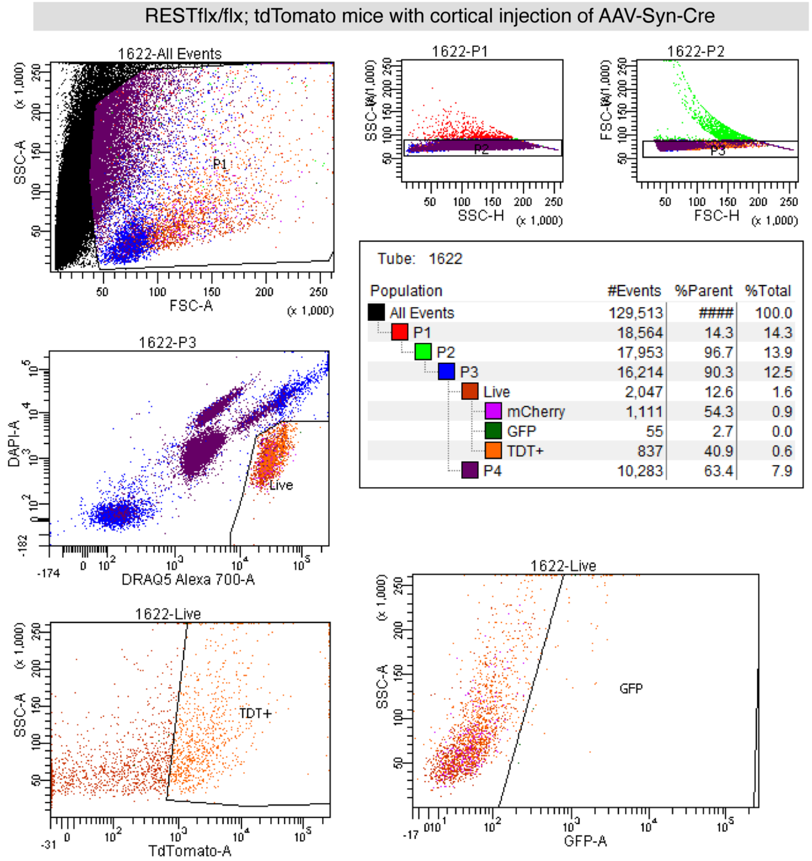

Tube: 1622

Population	#Events	%Parent	%Total
All Events	129,513	####	100.0
P1	18,564	14.3	14.3
P2	17,953	96.7	13.9
P3	16,214	90.3	12.5
Live	2,047	12.6	1.6
mCherry	1,111	54.3	0.9
GFP	55	2.7	0.0
TDT+	837	40.9	0.6
P4	10,283	63.4	7.9

Supplementary Fig. 11. Fluorescence activated cell sorting (FACS) gating strategies. Dissociated cortical neurons expressing AAV-Syn-GFP (wild-type) (**a**) or AAV-Syn-Cre (REST cKO) **(b)** were flow-sorted on a BD ARIA sorter using a 70 um nozzle and a sequential gating strategy. All events were first gated by SSH-H vs FSC-H to exclude cell debris. Live cells were isolated by manually gating for the ones with high DRAQ5 and low DAPI signal. Cells expressing GFP were further isolated from live cells by gating for high GFP and low tdTomato signals **(a)**, while cells expressing tdTomato (expression induced by AAV-Syn-Cre) were gated for low GFP and high tdTomato signals **(b)**.
