# Supplementary material for: Transcription factor network analysis identifies REST/NRSF as an intrinsic regulator of CNS regeneration in mice
Source: Nat Commun. 2022 Jul 29;13:4418. doi: 10.1038/s41467-022-31960-7 (PMC9338053; doi:10.1038/s41467-022-31960-7)
Supplement: Supplementary file 3 — Description of Additional Supplementary Files [file 41467_2022_31960_MOESM3_ESM.pdf]

Title: Supplementary Data 1.
Description: Differentially expressed genes (DEGs) comparing wild-type and REST knockout cortical motor neurons at 0, 1, 3, 7 days after SCI.

Title: Supplementary Data 2.
Description: Annotation of molecules in the regeneration-associated protein-protein interaction network in Figure 4D.

Title: Supplementary Data 3.
Description: Module eigengenes (MEs) of co-expression gene networks and module membership of each gene in RNA-seq of wild-type or REST knockout cortical motor neurons in sham or SCI conditions.

Title: Supplementary Data 4.
Description: Expression level changes of REST-repressed genes predicted by ARACNe comparing RGCs sorted at 1, 3, 5 days after optic nerve crush with pro-regenerative treatment to non-regenerative RGCs with control treatment.

Title: Supplementary Data 5.
Description: REST-targeted genes predicted by DNA footprinting analysis of ATAC-seq data generated in RGCs sorted at 0 (no crush), 1, and 3 days after optic nerve crush.
