# Supplementary material for: Transcription factor network analysis identifies REST/NRSF as an intrinsic regulator of CNS regeneration in mice
Source: Nat Commun. 2022 Jul 29;13:4418. doi: 10.1038/s41467-022-31960-7 (PMC9338053; doi:10.1038/s41467-022-31960-7)
Supplement: Supplementary file 9 — Reporting Summary [file 41467_2022_31960_MOESM9_ESM.pdf]

nature research

Corresponding author(s): Daniel Geschwind, Larry Benowitz

Last updated by author(s): May 9, 2022

Reporting Summary

Nature Research wishes to improve the reproducibility of the work that we publish. This form provides structure for consistency and transparency in reporting. For further information on Nature Research policies, see our Editorial Policies and the Editorial Policy Checklist.

Statistics

For all statistical analyses, confirm that the following items are present in the figure legend, table legend, main text, or Methods section.

n/a	Confirmed	
☐	☒	The exact sample size (*n*) for each experimental group/condition, given as a discrete number and unit of measurement
☐	☒	A statement on whether measurements were taken from distinct samples or whether the same sample was measured repeatedly
☐	☒	The statistical test(s) used AND whether they are one- or two-sided<br>*Only common tests should be described solely by name; describe more complex techniques in the Methods section.*
☐	☒	A description of all covariates tested
☐	☒	A description of any assumptions or corrections, such as tests of normality and adjustment for multiple comparisons
☐	☒	A full description of the statistical parameters including central tendency (e.g. means) or other basic estimates (e.g. regression coefficient) AND variation (e.g. standard deviation) or associated estimates of uncertainty (e.g. confidence intervals)
☐	☒	For null hypothesis testing, the test statistic (e.g. *F*, *t*, *r*) with confidence intervals, effect sizes, degrees of freedom and *P* value noted<br>*Give P values as exact values whenever suitable.*
☒	☐	For Bayesian analysis, information on the choice of priors and Markov chain Monte Carlo settings
☒	☐	For hierarchical and complex designs, identification of the appropriate level for tests and full reporting of outcomes
☐	☒	Estimates of effect sizes (e.g. Cohen's *d*, Pearson's *r*), indicating how they were calculated

Our web collection on statistics for biologists contains articles on many of the points above.

Software and code

Policy information about availability of computer code

Data collection	qPCR data - Roche LightCycler Software<br>Imaging data - Zeiss LSM 880<br>Sequencing data - Illumina HiSeq 4000 softwares
Data analysis	RNA-seq analysis: STAR v2.5.2b, picard tools v2.5.0, Salmon v0.7.2, Tximport v1.0.3, DEseq2 v1.26.0, GSEA v2.0, limma v3.30.13, R v3.3.0, WGCNA v1.51, g:Profiler v0.7.0, clusterProfiler v3.14.3, ggplot2 v2.2, igraph 1.2.4, DAPPLE (gene pattern public server), Ingenuity Pathway Analysis (IPA) Software (Qiagen).<br>ATAC-seq footprinting analysis: TOBIAS v0.9.0, samtools v1.11<br>Image analysis: Imaris 9.0; Image J (FIJI) v1.0.<br>Graphing and statistical analysis: GraphPad Prism v8<br>Only existing software tools were used for this study.

For manuscripts utilizing custom algorithms or software that are central to the research but not yet described in published literature, software must be made available to editors and reviewers. We strongly encourage code deposition in a community repository (e.g. GitHub). See the Nature Research guidelines for submitting code & software for further information.

Data

Policy information about availability of data

All manuscripts must include a data availability statement. This statement should provide the following information, where applicable:

- Accession codes, unique identifiers, or web links for publicly available datasets
- A list of figures that have associated raw data
- A description of any restrictions on data availability

RNA-seq data generated in this paper have been deposited in the Gene Expression Omnibus (GEO) with accession numbers GSE141583 and GSE142881. ATAC-seq data for footprinting analysis is part of our other study (90) with accession number GSE184547. There is no restriction in data availability.

Field-specific reporting

Please select the one below that is the best fit for your research. If you are not sure, read the appropriate sections before making your selection.

☒ Life sciences ☐ Behavioural & social sciences ☐ Ecological, evolutionary & environmental sciences

For a reference copy of the document with all sections, see nature.com/documents/nr-reporting-summary-flat.pdf

Life sciences study design

All studies must disclose on these points even when the disclosure is negative.

Sample size	Sample sizes were not predetermined based on statistical methods, but were chosen based on previous experiments with similar design (at least 3 independent biological replicates for each condition), which gave sufficient statistics for the effect size used （Yin et al., 2006; Yin et al. 2009; Chandran et al., 2016; Anderson et al., 2018).
Data exclusions	For RNA-seq data, we excluded genes that have zero count levels in 50% of samples analyzed. For experiment involving spinal cord or optic nerve injury, mice exhibiting incomplete crush were excluded for further analysis.
Replication	Each experiment presented in the paper was repeated in multiple mice (between 3 and 10 per experiment). Cell culture experiments were repeated for at least 3 times. All attempts at replication were successful.
Randomization	Mice with different litters, body weights and sexes were randomized and assigned to different treatment groups prior to downstream experiments, including RNA-seq, cell culture, surgery and immunohistochemistry.
Blinding	The investigators conducting in vivo experiment, imaging acquisition and analysis were blind to the identity of the cases. Blinding was not possible during quality control of RNA-seq data because the investigator needs to know sample identities to determine outliers in control or treated group.

Reporting for specific materials, systems and methods

We require information from authors about some types of materials, experimental systems and methods used in many studies. Here, indicate whether each material, system or method listed is relevant to your study. If you are not sure if a list item applies to your research, read the appropriate section before selecting a response.

Materials & experimental systems

n/a	Involved in the study	
☐	☒	Antibodies
☒	☐	Eukaryotic cell lines
☒	☐	Palaeontology and archaeology
☐	☒	Animals and other organisms
☒	☐	Human research participants
☒	☐	Clinical data
☒	☐	Dual use research of concern

Methods

n/a	Involved in the study	
☒	☐	ChIP-seq
☐	☒	Flow cytometry
☒	☐	MRI-based neuroimaging

Antibodies

Antibodies used	Primary antibodies: anti-GFAP (DAKO Z0334, 1:1000), anti-Synaptophysin (Synaptic Systems 101004, 1:1000), RFP (1:500, Abcam ab62341), streptavidin-HRP (1:300, PerkinElmer SAT704A001EA), anti-ATF3 (1:100, Abcam Ab207434), anti-SOX11 (1:500, Millipore ABN105), anti-pSTAT3 (1:200, Cell Signaling 9145), anti-pCREB (1:100, Alomone Labs), and anti-bIII tubulin [TUJ1] (1:500, Biolegend 801201), anti-beta-actin (1:5000, Sigma A5441 clone AC-15), anti-GAP-43 (1:500, Abcam, ab75810).<br>Secondary antibodies: goat-anti-rabbit 488 (1:500, thermofisher A32731), goat-anti-rabbit 555 (1:500, thermofisher A32732), goat-anti-rabbit 594 (1:500, thermofisher A32733), goat-anti-mouse 488 (1:500, thermofisher A11029), goat-anti-mouse 555 (1:500,

thermofisher A32727), goat-anti-mouse 647 (1:500, thermofisher A21235), anti-rabbit IgG, HRP-linked Antibody (1:2000, cell signaling 7074), anti-mouse IgG, HRP-linked antibody (1:2000, cell signaling 7076).

Validation

GFAP antibody was validated by the manufacturer for species specificity. This antibody has been verified by us and others by observing increased gliosis near lesion center of injured spinal cord; Synaptophysin antibody was verified by the manufacturer showing a single band in western blot; RFP antibody was verified by co-labeling with our tdTomato mice expressing RFP; ATF3 antibody was validated by the manufacturer in ATF3 knockout human cell line; Sox11 antibody was validated by the manufacturer in western blot of mouse brain showing a single band; pSTAT3 antibody was validated by the manufacturer in ChIP and western blot. GAP43 antibody was validated by immunoprecipitation and blotting of GAP43 in human cell line. TUJ1 and beta-actin antibodies are widely used and verified by multiple studies.

Animals and other organisms

Policy information about studies involving animals; ARRIVE guidelines recommended for reporting animal research

Laboratory animals

Details on the mice used in this study have been included in the Methods section of this manuscript. Mouse lines, including 129S1, C57BL/6J, loxP-REST-loxP (RESTflx/flx), B6.Cg-Tg(Thy1-CFP)23Jrs/J, and Rosa26-CAG-loxP-STOP-loxP-tdTomato (STOPflx/flx TdTomato), were purchased from Jackson Laboratory. RESTflx/flx; tdTomato homozygous mice were generated by crossing RESTflx/flx (107) and STOPflx/flx TdTomato mice. Young adult mice between 4-6 weeks old including both sexes were used for all experiments in spinal cord studies and 8 -12 week old animals in optic nerve regeneration studies. Mice were bred and raised under a 24-h light-dark cycle with 12 h of light and 12 h of darkness. Ambient temperature was maintained at 22 °C ± 2 °C and humidity between 40-60%.

Wild animals

No wild animals were used in this study

Field-collected samples

No field-collected samples were collected in this study.

Ethics oversight

Experiments performed at University of California, Los Angeles were approved by the Animal Research Committee of the Office for Protection of Research Subjects. Experiments performed at Boston Children's Hospital were approved by the Institutional Animal Care and Use Committee (IACUC).

Note that full information on the approval of the study protocol must also be provided in the manuscript.

Flow Cytometry

Plots

Confirm that:

☒ The axis labels state the marker and fluorochrome used (e.g. CD4-FITC).

☒ The axis scales are clearly visible. Include numbers along axes only for bottom left plot of group (a 'group' is an analysis of identical markers).

☒ All plots are contour plots with outliers or pseudocolor plots.

☒ A numerical value for number of cells or percentage (with statistics) is provided.

Methodology

Sample preparation

Fluorescence-activated cell sorting (FACS) was performed to purify fluorescece-labeled neurons dissociated from mouse sensorimotor cortex or retina. A detailed description of of sample preparation procedure is included in the Methods sections 'FACS isolation of adult cortical motor neurons' and 'FACS isolation of retinal ganglion cells'.

Instrument

Cell sorting was performed using BD FACS Aria II.

Software

BD DIVA v8
FlowJo software (Tree Star Inc.)

Cell population abundance

We obtained ~2,000 neurons sensorimotor cortex per mouse, and ~2,000 - 10,000 retinal ganglion cells per mouse retina. Cell purity post-sorting was checked periodically by examining the percentage of fluoresently labeled cells using a fluorescent microscope.

Gating strategy

Cells were selected for live cell scatter in FSC/SSC then for singlets in FSCa/FSCh or FSCh/FSCw and SSCh/SSCw then for DAPI negativity and DRAQ5 positivity (dead cell exclusion). Other markers were determined positive when signal was above FMO (fluorescence minus one) control.

☒ Tick this box to confirm that a figure exemplifying the gating strategy is provided in the Supplementary Information.
